# Lasting deficit in inhibitory control with mild traumatic brain injury

**DOI:** 10.1038/s41598-017-14867-y

**Published:** 2017-11-02

**Authors:** Benjamin Xu, Marco Sandrini, Sarah Levy, Rita Volochayev, Oluwole Awosika, John A. Butman, Dzung L. Pham, Leonardo G. Cohen

**Affiliations:** 1 Human Cortical Physiology and Neurorehabilitation Section, National Institute of Neurological Disorders and Stroke, National Institutes of Health, Bethesda, MD 20892 USA; 2 Center for Neuroscience and Regenerative Medicine, Uniformed Services University of Health Sciences, Bethesda, MD 20814 USA; 3 Radiology and Imaging Sciences, Clinical Center, National Institutes of Health, Bethesda, MD 20892 USA

## Abstract

Being able to focus on a complex task and inhibit unwanted actions or interfering information (i.e., inhibitory control) are essential human cognitive abilities. However, it remains unknown the extent to which mild traumatic brain injury (mTBI) may impact these critical functions. In this study, seventeen patients and age-matched healthy controls (HC) performed a variant of the Stroop task and attention-demanding 4-choice response tasks (4CRT) with identical stimuli but two contexts: one required only routine responses and the other with occasional response conflicts. The results showed that mTBI patients performed equally well as the HC when the 4CRT required only routine responses. However, when the task conditions included occasional response conflicts, mTBI patients with even a single concussion showed a significant slow-down in all responses and higher error rates relative to the HC. Results from event-related functional magnetic resonance imaging (efMRI) revealed altered neural activity in the mTBI patients in the cerebellum-thalamo-cortical and the fronto-basal-ganglia networks regulating inhibitory control. These results suggest that even without apparent difficulties in performing complex attention-demanding but routine tasks, patients with mTBI may experience long-lasting deficits in regulating inhibitory control when situations call for rapid conflict resolutions.

## Introduction

The ability to focus on a complex task and suppress interfering information or unwanted response quickly (i.e., inhibitory control) are essential cognitive functions for carrying out daily and other important activities (e.g., driving a car, crossing busy streets, or patrolling in a combat zoon). Patients with severe traumatic brain injury (TBI) typically show symptoms of various cognitive difficulties including memory, attention, inhibitory control, and other executive functions^1,2^. These difficulties often co-occur with extensive brain lesions or with significant difficulties in maintaining task-relevant attention^3,4^. Recent studies also showed that potential functional and structural abnormalities of the brain may occur and persist after acute mild TBI (mTBI)^5–8^. mTBI accounts for about 80% of all traumatic brain injuries in the United States^9^. However, it remains a question whether patients with chronic mTBI may also suffer from long-lasting impact on cognitive functions, particularly in maintaining (i.e., sustained) attention and inhibitory control.

Current understanding of basic but critical cognitive functions such as sustained attention and inhibitory control with mTBI is limited. Previous studies investigating attention deficits with TBI often included patients with severe TBI or did not take into account the potential interaction between attention deficits and the ability to carry out tasks that also required inhibitory control^3,4,10,11^. A few studies reported impairment in response inhibition with mTBI patients but lacked experimental control for potential deficits in sustained attention and strategy-induced differences in brain activity^12–14^. There is strong evidence that the attention system and the inhibitory control system engage neural networks with some shared circuits/regions but different dependence on the fronto-basal-ganglia network^15–17^. The attention system has been shown to engage the cingulate and frontoparietal network^15^, while the right inferior-frontal cortex (rIFC Pars Opercularis), supplementary motor area (preSMA), and their connections between and to the basal ganglia (e.g., the subthalamic nuclei) are particularly important for rapid inhibitory control^17,18^. Although significant deficits in attention including sustained attention would likely impair the performance of cognitive tasks in general^19^, lesion studies showed that damage in the right preSMA or rIFC was associated with impairment in rapid response inhibition but not with the degradation of task responses in general^20–22^. However, substantial evidence suggests that concussion (i.e., a brief loss of consciousness) induced by a closed-head impact is likely caused by a sudden abnormal discharge of neuronal activity both in the cortical and subcortical systems (e.g., the reticular activating system or RAS) although the exact mechanism remains unclear^23^. The extent of structural injuries at the cellular, cortical, and subcortical level, and their white matter connections is also difficult to assess after mTBI even with the most up-to-date technologies^24–28^. The majority of the patients with mTBI often appear asymptomatic post-injury after a recovery period of a few days or weeks and able to carry on cognitive tasks normally^29–31^. However, the later must be viewed with caution. Normal cognitive performance assessed in clinical settings may be confounded with differential cognitive effort/strategies for specific tasks relative to the non-TBI controls and, therefore, significant differences in brain function^29^. We hypothesized that cognitive functions such as rapid inhibitory control and sustained attention that differentially rely on these brain systems may bear substantial impact. Recent findings indicate that the impact of mTBI on functional recovery may have been significantly underestimated^5–8^.

We examined the ability of patients with chronic mTBI and age-matched healthy controls (HC) to perform tasks that required sustained attention with or without significant burden on inhibitory control. We also measured changes in brain activity associated with these tasks. Seventeen predominantly mTBI patients (14 mild and 3 moderate; average time [month] since last concussion = 28 [±28.9]) and 17 HC completed the study (see Table S1 in the Supplementary Material for the list of the TBI patients). The patients did not differ significantly from the HC in performing working memory, attention, and simple motor movement tasks (see Table S2 in the Supplementary Material). For the experimental task, all participants performed a 4-choice response task (4CRT) under three conditions with identical stimuli during functional magnetic resonance imaging (fMRI). They were instructed to make choice (i.e., “go”) responses with one of four buttons corresponding to four stimulus orientations (e.g., pressing the left button for a leftward-pointing arrow). One of the task conditions required only the “go” responses (All-Go condition), and the other two required, occasionally, either responding with a button opposite to the stimulus orientation (Switch condition) or stopping a response when a stop-signal appeared (Stop condition). Participants also performed, on a separate day, a variant of the Stroop task examining the ability to suppress interfering information as an additional task for measuring the inhibitory control function. (See Methods for details and the rationale of the task design).

## Results

**Figure 1:**
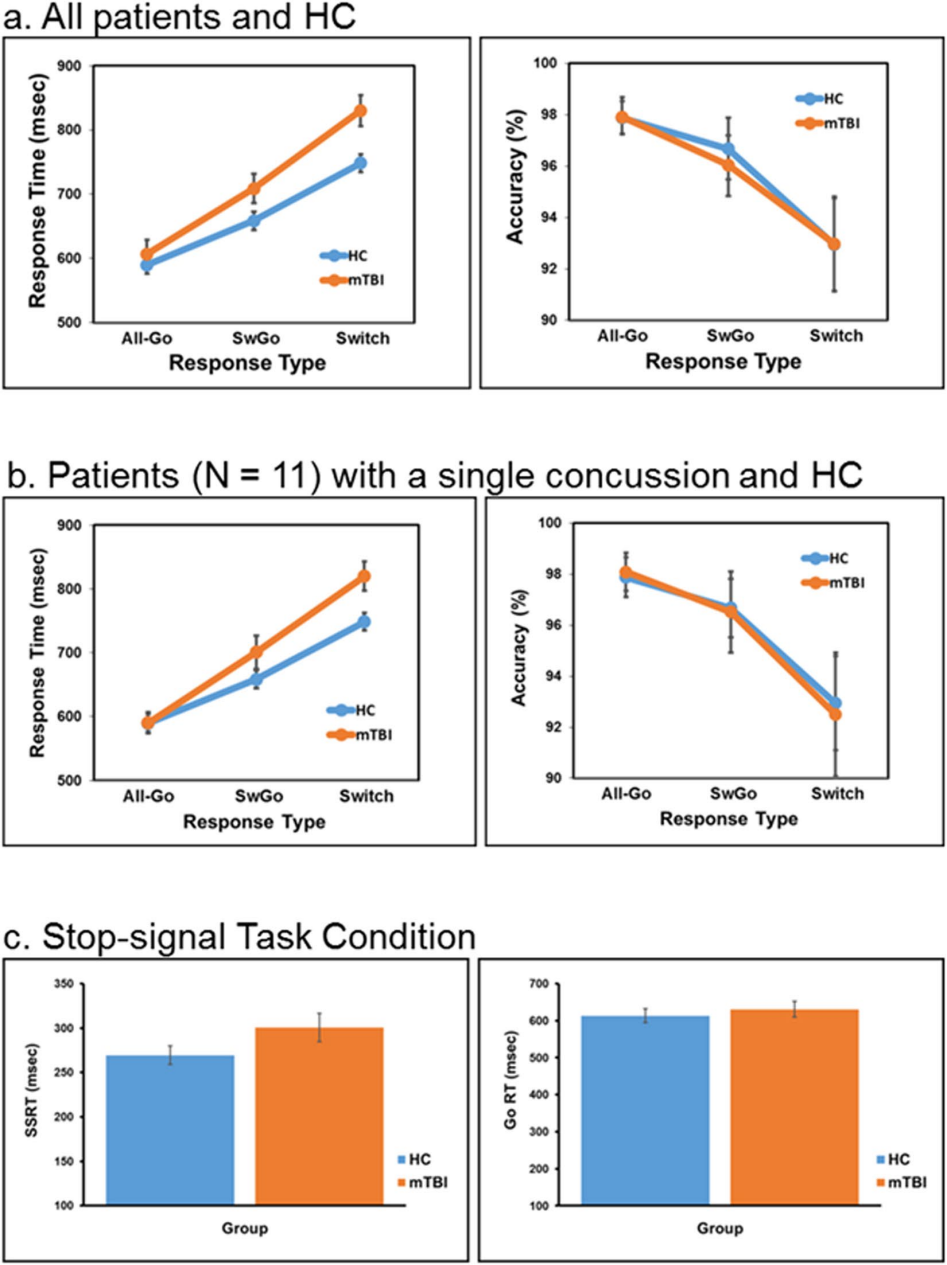
Results of the 4CRT. All three task conditions required continuous monitoring (i.e., sustained attention)^15^ of the stimulus type in order to make a quick and accurate response choice. (**a**) Shows when the task condition required only the primary “go” responses (i.e., the All-Go condition), the mTBI group (with single or multiple concussions) performed equally well as the HC group in both response time (RT) (mTBI = 607 ms [±90], HC = 590 ms [±58]) and accuracy (mTBI = 99% [±2.6], HC = 97.9% [±3.2]). However, when inhibiting the primary “go” response was required for a button-switch response on some of the trials (i.e., the response “Switch” condition), patients showed a significant slowdown in all RTs including the “go” (SwGo) response (709 ms [±91]) and the “Switch” response (830 ms [±97]) relative to the HC (“SwGo” RT = 658 ms [±58], “Switch” RT = 749 ms [±58]; t_(31)_ = 1.90, p < 0.05; t_(31)_ = 2.97, p < 0.01). A Mixed Repeated-Measures ANOVA revealed a significant interaction between Group and Response Type (F_(2,62)_ = 224.12, MSe = 1352, p < 0.0001). The interaction was mainly due to the significantly longer RT of the patients in the Switch condition with both the “SwGo” and “Switch” responses comparing to the HC. (**b**) Shows similar patterns of results of the 4CRT when patients with multiple concussions were excluded (see Figure S1 in Supplementary Material for more patient results and statistics). **(c)** Results of the Stop-signal task condition. For this condition, participants were instructed to stop the primary “go” response when a “stop-signal” appeared occasionally. Similarly, patients showed a tendency of slowing in the stop-signal response time (SSRT), a critical measure of inhibition efficiency, relative to the HC (mTBI = 300 ms [±16], HC = 269 ms [±11], t_(20)_ = 1.63, p < 0.06). This task condition was analyzed separately from the other two conditions because only 11 healthy controls and 11 patients reached the expected level of performance (i.e., about 50% accuracy on the “stop” response) required for estimating the SSRT^55,56^. Notes: All-Go = “go” response in the All-Go task condition; SwGo = “go” response in the Switch condition.

The results showed that mTBI patients performed similarly as the HC in the 4CRT when only the “go” responses were required. This result is consistent with a recent report of mTBI patients using a choice reaction time task^32^. However, when inhibiting/stopping the primary/routine “go” response was necessary occasionally (i.e., in the Switch or Stop condition), patients had significantly longer response time (RT) relative to the HC (Fig. 1a and c). Similar results were observed even when patients with only a single reported concussion were included in the analysis (Fig. 1b).

**Figure 2:**
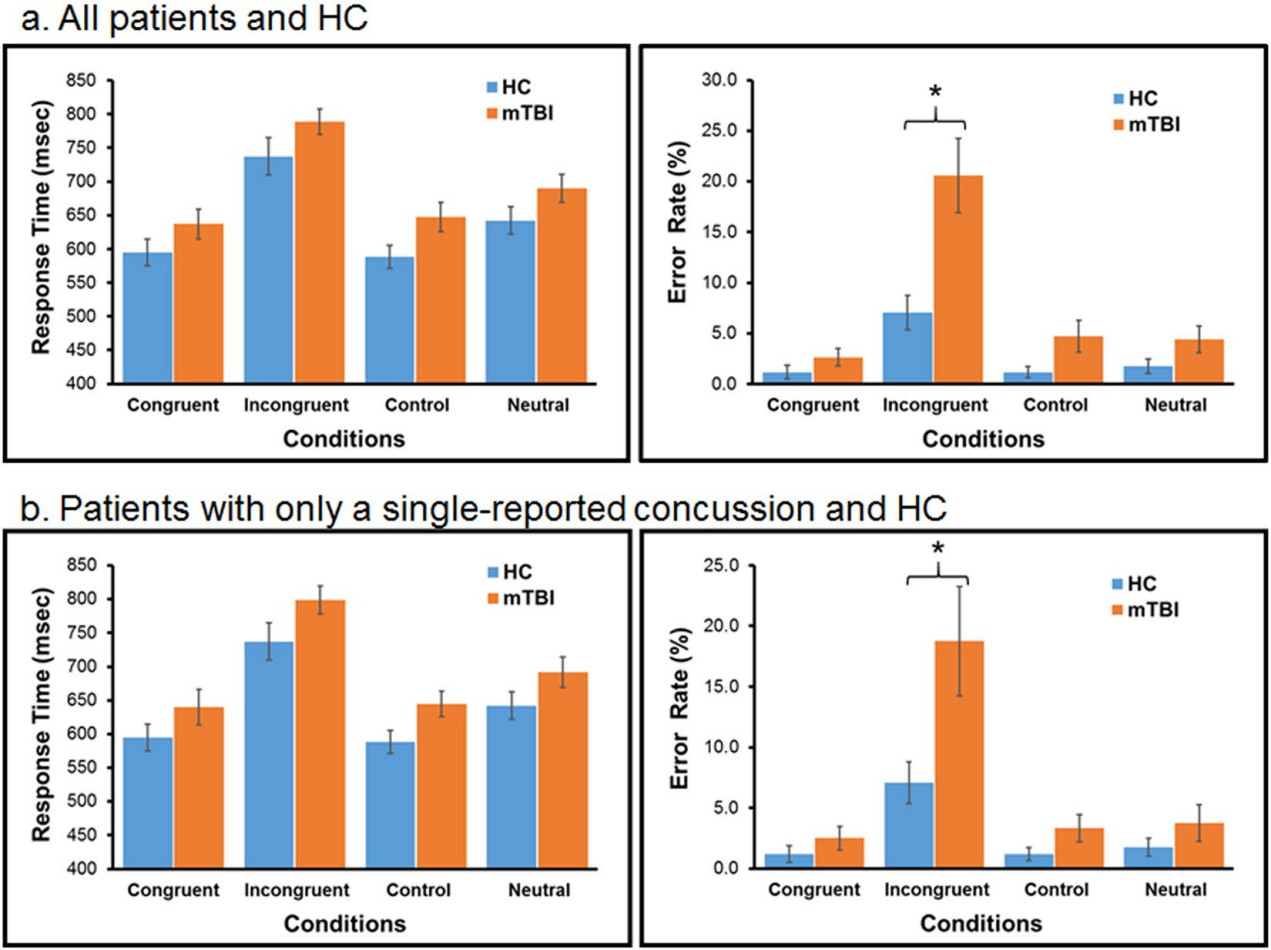
Results of the Stroop Task. (**a**) This analysis included all mTBI patients and HC. The results showed that patients had significantly more difficulty than HC in the Incongruent condition. There was a significant group difference in overall error rates (mTBI = 8.1%, HC = 2.8%; F_(1,96)_ = 11.58, MSe = 36.85, p < 0.002), and a significant Group by Condition interaction (F_(3,96)_ = 7.12, MSe = 36.85, p < 0.001). The interaction was mainly due to the significant group difference (Post hoc Tukey tests with p < 0.05) in the error rate with the incongruent words. Patients made almost three times more errors (20.6%) than HC (7.1%) in the Incongruent condition. Patients also tended to be slower overall than HC in RT (mTBI = 691 ms; HC = 641 ms) although the difference was not statistically significant (F_(1,96)_ = 3.14, MSe = 1268, p < 0.09) (also see Table 3 in the Supplementary Material). (**b**) Results from the 12 patients with only a single concussion. These results did not change substantially relative to those when all the patients were included in the analysis (group difference: F(_1,27_) = 8.29, p < 0.01; group x condition interaction: F(_3,81_) = 5.03, p < 0.003). (see Tables S2–S3 in Supplementary Material for more patient results).

These results were consistent with that of the Stroop task which required naming the ink color of words presented either in the color that matched the word names (i.e., congruent condition, e.g. “green” written in green color) or did not match (i.e., incongruent condition, e.g. “yellow” written in red color). These words were mixed with an equal number of neutral words (e.g., “real” written in one of the five colors) and controls (“****” written in one of the five colors). In order to name the color of the incongruent words quickly and correctly, participants must ignore or suppress the interfering information (i.e., the name of the words). The results showed that mTBI patients were less efficient in suppressing the interfering word name in the Incongruent condition than the HC. Patients made almost three times more errors than the HC in the Incongruent condition and had longer RT overall (Fig. 2a). Again, these results did not change substantially even when only patients with a single reported concussion were included in the analysis (Fig. 2b).

Overall, the behavioral results indicated that mTBI patients had significant difficulty in inhibitory control but with relatively intact attention system. As the results showed, mTBI patients had significant difficulties making responses quickly and accurately when inhibition of the primary stimuli (or interfering information) was required, irrespective of the nature of tasks (i.e., either making visual-motor responses in the 4CRT or naming words in the Stroop task). This difficulty cannot be simply attributed to a deficiency in the ability to maintain task-relevant attention in this study. The mTBI patients performed equally well as the HC during the 4CRT when additional demands in inhibitory control of responses were not required, despite of the attention-demanding nature of the task (i.e., continuous monitoring of the varying stimuli and making a “go” response with four different choices of the response buttons). Instead, the behavioral results showed a deficit in patients in performing tasks that required the suppression/inhibition of a primary response or interfering stimulus.

**Figure 3:**
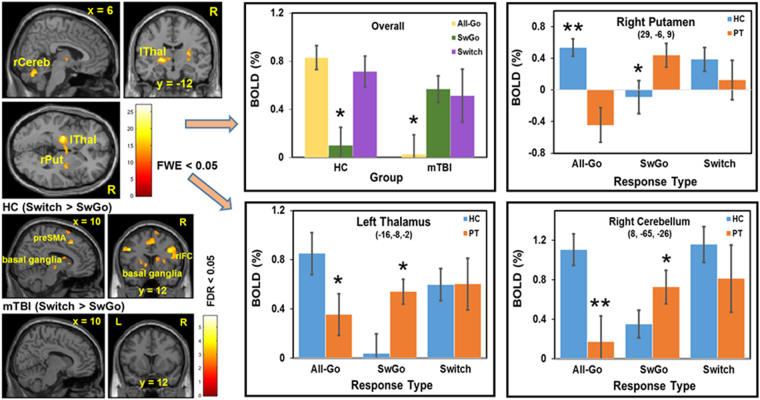
Results of efMRI brain activation. The top left images of whole-brain analysis showing a significant interaction between Group (mTBI and HC) and Response Type (All-Go and SwGo) at the second level analysis using the flexible factorial design with a Mixed Repeated Measures ANOVA in SPM12 (voxel-level uncorrected threshold p < 0.001, cluster threshold FWE p < 0.5). The bar graphs showed results of the significant clusters (3mm radius centered on the peak voxel) extracted from the “All-Go,” “SwGo,” and the “Switch” responses of both mTBI and HC groups. The top left bar graph is the average of all three clusters and ANOVA results (*post hoc Tuckey p < 0.05). The other 3 bar graphs show the results of planned t tests (**p < 0.01; *p < 0.05). The bottom left four images showed significantly more activation only in the HC group in the fronto-basal-ganglia inhibitory network during the “Switch” relative to the “SwGo” responses. A binary regions-of-interest (ROI) mask of the fronto-basal-ganglia network (i.e., LM1, rIFC, SMA, preSMA, and the basal ganglia) was applied in the analysis. Note: All-Go = “go” response in the All-Go condition; SwGo = “go” response in the Switch condition; Switch = “Switch” response in the Switch condition; lThal = left thalamus; rCereb = right cerebellum; rPut = right putamen.

To understand the performance differences and their relation to changes in brain function, we examined the brain activity of the participants performing the 4CRT. The fMRI results revealed a significant difference of brain activation in the response-control system involving the cerebello-thalamo-cortical (CTC) pathways (Fig. 3)^33,34^. The mTBI patients showed significantly (FWE p < 0.05) less activation than the HC in the left thalamus, right putamen, and right cerebellum when inhibiting the primary/routine “go” response was not required (in the All-Go task condition). The pattern of activation in these regions reversed when the task condition required inhibition of the primary “go” responses occasionally in the Switch condition. Unlike the patients who showed similar hyperactivation in the Switch condition for both the “go” and the “Switch” responses, the HC activated these regions differentially, that is, significantly less activation for the “go” than the “Switch” responses. (Fig. 3). The patients also did not show significant difference in brain activation between the “go” and “Switch” responses in the fronto-basal-ganglia regions (Fig. 3; also see Figure S2 in the Supplementary Material), a brain network critical for rapid response inhibition^17,35^. In addition, relative to the “go” responses in the All-Go condition, patients recruited more brain regions, including the rIFC (a node of the fronto-basal-ganglia inhibitory network), than the HC during the “go” responses when inhibition of the primary “go” stimuli was required occasionally (i.e., in the Switch and Stop-signal task conditions) (see Figures S3 and S4 in the Supplementary Material). The lack of significant differences of brain activation in the response inhibition and control networks between the “go” and “Switch” responses, and the recruitment of the rIFC during the “go” responses in the Stop-signal condition suggest that mTBI patients had difficulty differentially gating the “go” (a primary/routine action) and the “Switch” or “Stop” (sudden change of action) responses when the primary “go” responses must be suppressed occasionally. Instead, they appeared to heighten the inhibitory control system indiscriminately for all responses, consequently, further slowing down on all actions.

## Discussion

The significant differences between the mTBI patients and the HC in behavioral performance and brain activation suggested a significant functional alteration in inhibitory control with mTBI even when significant deficit in sustained attention was not observed. The significant differences in the activation of the CTC and the fronto-basal-ganglia systems between the mTBI patients and the HC indicate altered functioning of these brain regions. The CTC pathways have been shown to be critical to cognitive control of both attention and motor responses^33,34,36^. These subcortical structures and their connections within and to cortical regions are vulnerable to closed-head injuries like TBI^37–39^. The fronto-basal-ganglia, particularly, the rIFC has been shown to be critical for inhibitory response control^17^. Closed-head impact that causes the lost of consciousness may also affect the reticular-activating system (RAS) in the brain^23^ that controls arousal and interacts with the attentional systems^1,40^. Although the exact interaction between the CTC, RAS, and the fronto-basal-ganglia during inhibitory control remains to be understood, it has been shown that task-induced activity in the thalamus is influenced by the input from these systems^41^. mTBI patients may have altered ability to regulate the neuronal activity within these systems, especially, in situations when rapid suppression of a primary response or interfering information is necessary.

A closed-head impact that is sufficient to cause the loss of consciousness, even though brief, may exert enough force not only to disrupt brain functions but, in some cases, with long-lasting altered structural and functional normality. The behavioral and brain activation data from this study suggest that mTBI patients had difficulty differentially gating the “go” (a primary/routine action) and the “Switch” or “Stop” (a sudden change of action) responses when the primary “go” responses must be suppressed occasionally. In order to cope with the deficiency, neural activity in the fronto-basal-ganglia inhibitory control system becomes overly heightened for all responses so that the primary “go” response could be successfully suppressed when sudden change of action is needed. In other words, mTBI patients may have to mobilize the inhibitory control system indiscriminately for all responses in potential conflict situations. This altered activity in the CTC and fronto-basal-ganglia response inhibition network may lead to a burden on cognitive resources and a slowdown of all responses/actions. Unlike more severe TBI patients^1,3^, the attentional systems may be relatively intact in mTBI patients with sufficient system-regulation ability to allow relatively normal performance even with challenging and attention-demanding tasks so long as only routine actions are needed (e.g., performing a task in the All-Go condition). However, this ability breaks down when rapid inhibitory control becomes critical as in real life situations (e.g., daily driving, crossing busy streets, or patrolling in a combat zoon).

Although it is unclear why with mTBI, inhibitory control was more impaired than sustained attention in this case, we speculate that functional connectivity and likely structural integrity between the basal ganglia and cortex are sensitive to the concussive force even when there is no apparent structural injury. Recent findings have shown that subcortical regions such as the brainstem, corpus callosum, subcortical parasagittal white matter with connections to the basal ganglia, thalamus, and cerebellum are particularly susceptible to TBI^24,42,43^. Even with mild TBI, potential functional and structural abnormalities of the brain, though difficult to detect with routine clinical examinations, may persist^5,7,8^. As the fronto-basal-ganglia and the CTC networks are critical to rapid response inhibition or inhibitory control in general, functional disruption or injuries to white matter connections within these neural networks may selectively impair their efficiency in regulating rapid inhibitory responses. Such deficits in regulating inhibitory control when situations or tasks called for rapid conflict resolutions may be long lasting with mild traumatic brain injury.

## Methods

### Participants

Seventeen patients (14 with mild traumatic brain injury [mTBI] and three moderate TBI: 12 male and 5 female; mean age = 29.6 [±5.1]) and 17 non-TBI age-matched healthy control (HC) volunteers (6 male and 11 female, mean age = 28.3 [±4.2]) participated in the study. All participants had a normal neurological examination, with normal or corrected vision, and were right-handed based on the evaluation with the Edinburgh Handedness Inventory^44^. All HC participants’ routine structural MRI scans were also normal. Only three of the 17 patients were clinically diagnosed as with moderate TBI (excluding these patients did not change overall results and statistical outcomes. See results discussion below). Twelve of the mTBI patients reported only one concussion. Seven patients showed limited focal lesions in routine structural MRI scans that did not involve the CTC, RAS, and the fronto-basal-ganglia response-inhibition network (see Table S1 in the Supplementary Material). All participants had a baccalaureate or higher level of education except for one patient who completed high school. In addition to the neurological examination, TBI patients also received a detailed clinical examination which included medical history, use of medications, the Mini-mental State Examination (MMSE), and the Beck Depression Inventory II (BDI-II). No patients were taking medications nor had a more-than-minimal BDI-II score (i.e., >19) at the time of the study. Other exclusion criteria included alcoholic or drug addition, chronic use of medications acting primarily on the central nervous system, BDI-II score greater than 29, pregnancy, history of epilepsy, and less than three months post-TBI. All concussions were resulted from daily activities or motor-vehicle accidents. All participants gave an informed and signed written consent. All experimental protocols were approved by the Combined Neuroscience Institutional Review Board at the National Institutes of Health for participating in the study and in accordance with the Declaration of Helsinki as well as other relevant guidelines and regulations. Participants received monetary compensation for their time in the study.

### Task materials and procedure

#### Baseline measures

All participants completed a visual digit-span test and four sub-tests from the Automated Neuropsychological Assessment Metrics (ANAM, developed by the U.S. Department of Defense): Code Substitution for short-term memory (CSM); Matching to Sample for visual spatial memory (M2S); 1-back Continuing Performance for memory and attention (CP); Pursuit Tracking for visual motor and attention (PurT). The ANAM sub-tests were administered via the ANAM software (created by the University of Oklahoma) on a Windows personal computer. There were no statistically (two-sample t tests) significant differences between the mTBI patients and HC groups (see Table S2 in Supplementary Material).

#### 4-choice response task (4CRT)

**Figure 4:**
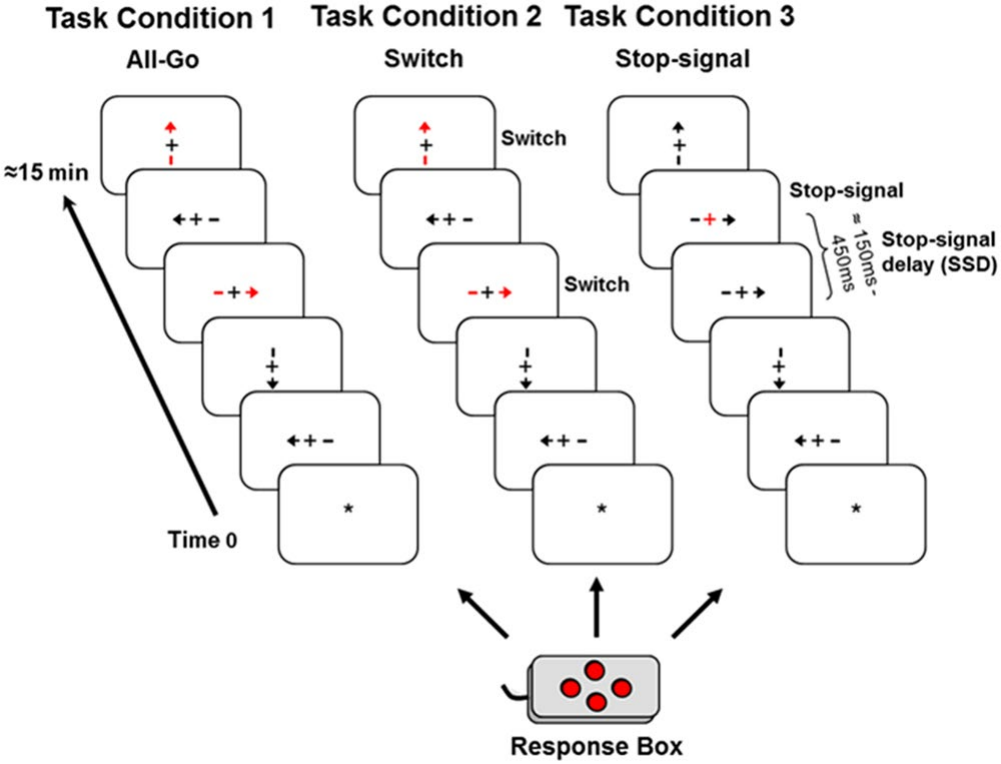
Design of the 4-choice response task (4CRT). Task Condition 1 (All-Go) included only the “go” responses (i.e., All-Go) with a total of 80 trials. Task Condition 2 (Switch) had identical stimuli as those in Condition 1 (All-Go). The only difference was that participants were told to press the opposite response button (i.e., a “Switch” response) relative to the stimulus orientation when they saw a red arrow (40 out of 160 trials). Task Condition 3 (Stop-signal) was a variant of the stop-signal task (SST). It included a delayed visual-cue (i.e., the stop-signal) to signal a “Stop” response. The primary “go” trials were identical to Condition 1 and 2 except that no red arrows were presented and for 40 (25%) out of the 160 trials, the “+” sign in the middle of the stimulus would turn red (the stop-signal) with a variable stop-signal delay (SSD) after the onset of the stimulus.

All participants performed the 4CRT during an event-related fMRI (efMRI) session. The 4CRT has been described and reported in detail in our previous publication^45^. The stimuli consisted of arrows with four orientations (up, down, left, and right) and a fixation point “ + ” in the middle (see Fig. 4). All four orientations had an equal probability of occurrence in three task conditions (see below). The arrow stimuli were presented one at a time using the E-Prime software (by Psychological Software Tools, Inc) for a duration of 1500 msec or until a response was made. The data collection period was 2000 msec for each trial. The stimulus dimension was maintained at less than 2 degrees of visual angle relative to the subject’s viewing position inside the MR scanner. The inter-stimulus-interval (ISI) was jittered for the efMRI design with an average ISI of about four seconds (range 2–6 seconds) yielding a stimulus-onset-asynchrony (SOA) between 3.5 and 7.5 seconds. There were also six 10-second ISIs (the Rest period) interspersed within each scan run. Prior to the stimulus onset, there was a fixation point (a yellow star “*”) in the center of the display. At the offset of each stimulus, the fixation point appeared and stayed on until the onset of the next trial (the 10-second Rest period was indicated with an uppercase letter “R”). A four-button response box (see Fig. 4) was configured such that the top, bottom, left, and right buttons corresponded to the four stimulus orientations. The response buttons were situated with equal distance around a center space (about 1 cm^2^). All responses were made with the right index finger. The primary response (i.e., the “go” response) required pressing the button consistent with the arrow orientation. Participants were instructed to always place their right index finger in the center space on the box between responses. They performed the task in three experimental conditions with identical “go” responses.

Task Condition 1 (All-Go): This condition included only the “go” responses (i.e., All-Go) with a total of 80 trials. All but 20 trials (25%) appeared in red color and the rest in gray. Participants were told explicitly that color was irrelevant for this task condition and that they should press the corresponding button as quickly as possible without sacrificing response speed for accuracy. The All-Go condition served two purposes: (1) to provide a baseline measure of the primary “go” response performance (i.e., RT and accuracy) when only “go” responses were required; (2) to establish a consistent stimulus-response association for the context of the response Switch condition (described below).

Task Condition 2 (Switch): This condition had identical stimuli as those in Condition 1 (All-Go). The only difference was that participants were told to press the opposite response button (i.e., a “Switch” response) relative to the stimulus orientation when they saw a red arrow (40 out of 160 trials). For example, participants were instructed to press the left button when a right-pointing red arrow appeared. This manipulation placed the burden on suppressing/inhibiting an impulse/tendency for the primary “go” (i.e., dominant) response in order to initiate a “Switch” response. It is important to emphasize that as it was designed, the response component of the “Switch” trials was identical to that of the primary “go” responses and with the same extent of practice (or over-learning) in terms of pressing the four response buttons. What differed was the need to inhibit the primary but undesirable “go” process in the Switch condition and generate a “Switch” response.

Task Condition 3 (Stop-signal): The Stop-signal condition was a variant of the stop-signal task (SST)^46^. It included a delayed visual-cue (i.e., the stop-signal) to signal a “Stop” response. The primary “go” trials were identical to Condition 1 and 2 except that no red arrows were presented and for 40 (25%) out of the 160 trials, the “+” sign in the middle of the stimulus would turn red (the stop-signal) with a variable stop-signal delay (SSD) after the onset of the stimulus. Participants were instructed to withhold/stop their response as soon as the stop-signal appeared. The SSD was set at 150 msec for the first “Stop” trial and, then a staircase tracking method^47^ was implemented such that for every successfully-stopped (i.e., Stop-inhibit) response, the SSD was increased by 50 msec to make it harder to stop on the next trial, and for each fail-to-stop (i.e., Stop-respond) trial, the SSD decreased by 50 msec. The longest possible SSD was 450 msec. Participants were told explicitly at the beginning of the task condition that only a few trials would have the stop-signal and that it was expected that they would not be able to withhold the response for many of those trials. As it was for the other two task conditions, participants were instructed repeatedly that they should respond as quickly as possible for all trials and should not sacrifice response speed for accuracy. The dynamic SSD control method resulted in an overall Stop-inhibit rate of about 50% for 11 HC (52.7% [±2.1]) and 11 PT (52% [±2.4]), which was close to the ideal rate of 50%^47^. Only these participants from both groups were included in the final analysis for the Stop-signal task condition.

The design of the 4CRT used identical button-press responses between task conditions. The key difference between the All-Go and the Switch or Stop task conditions was that the All-Go condition did not require the inhibition of an on-going motor response (i.e., the primary “go” response), while the Switch and Stop-signal conditions required the inhibition/stopping of the primary “go” response for 25% of the trials. Task Condition (All-Go, Switch, and Stop-signal) and Response Type (“All-Go,” “SwGo,” “SSTGo,” “Stop-inhibit,” and “Stop-respond”) were within-subject factors (SwGo = the “go” responses in the Switch condition; SSTGo = the “go” response in the Stop-signal condition; Stop-inhibit = successfully stopped response; Stop-respond = failed-to-stop response). All participants were given the three task conditions within a single fMRI session. The All-Go condition was always presented first with the Switch and Stop task conditions counterbalanced between subjects. All participants were given practice trials at the beginning of the experiment and sufficient time for practicing or getting familiar with the response box. During the experiment, the response box was fixed on the right side of the participant so that it was stationary and no firm hand gripping on the box was necessary.

#### Stroop Task

The design of the Stroop task included four types of stimuli printed in five different colors (i.e., red, yellow, blue, green, and purple). The four types of stimuli were composed of: (1) color words printed in the color that matched the word name (i.e., the Congruent condition, e.g. “green” written in green color); (2) color words printed in the color that was different from the word name (i.e., the Incongruent condition, e.g. “yellow” written in red color); (3) non-color words (i.e., the Neutral condition, e.g., “real”); and (4) stars ***** (i.e., the Control condition). The stimuli for the Neutral and Control conditions were printed randomly in the five different colors. Each of the four types of stimuli had equal number (20 per type) of trials (total = 80 trials) and were semi-randomly distributed in a single test list. The participants were instructed to name the color of the stimuli as quickly and accurately as possible. The stimuli were presented via the E-Prime software (by Psychological Software Tools, Inc) with the voice-key input. The stimulus-onset asynchrony (SOA) was 2000 ms. Participants were seated in a fixed distance that assured a less than two-degree visual angle of the stimuli. Misnaming of the color or hesitation in pronunciation (i.e., light aspiration or delayed naming until after the onset of the subsequent stimulus) was counted as an error. All participants were given a short practice session prior to the experimental session.

The current design of the study allowed us to make the following predictions. All things being equal, relative to the HC group: (1) significant impairment (or deficit) in sustained attention with mTBI would result in poor performance (i.e., longer response time and/or higher error rates) in all task conditions; (2) significant deficits in both sustained attention and inhibitory control would render worse performance for the “go” response in the Switch and Stop conditions that required rapid inhibitory control than that in the All-Go condition, in addition to poor performance in all conditions; (3) Significant deficit in inhibitory control alone with relatively intact attention system would result in similar performance in the All-Go condition which did not require a significant burden on inhibitory control; and (4) significant differences in cognitive strategies with mTBI would result in differences in activation of brain networks even with the same task conditions and similar performance. Similarly, deficit in inhibitory control with mTBI would result in significantly more difficulty in performing the Stroop task than the HC when suppressing interfering information was needed.

### Behavioral data analysis

For the Digit Span and the four sub-sets of the ANAM tests, two-sample t tests (two-tailed) were carried out for each test. The RT and accuracy data were analyzed separately. For the 4CRT, the All-Go and the Switch conditions were combined using Mixed Repeated-Measures ANOVA (mRMANOVA) with Group (PT and HC) as a between-subject factor and Response Type (“All-Go,” “SwGo,” and “Switch” responses) as a within-subject factor. One PT did not perform the Switch condition correctly and was not included in the final analysis. The Stop-signal task condition was analyzed separately because the “go” response tended to be influenced by the stop-signal trials^45,48,49^ and only 11 participants in each group met the 50% criteria for the “Stop” response (see discussion above). Two-sample t tests were carried out separately for the SSRT and the RT of the SSTGo (i.e., the “go” response in the Stop-signal task [SST] condition). For the Stroop task, mRMANOVA was carried out for the RT and ACC (accuracy) separately with Group (HC and PT) as a between-subject factor and Condition (Congruent, Incongruent, Control, and Neutral) as a within-subject factor. All RTs ≥ two standard deviations of the mean of a task-condition within each subject in the 4CRT were considered as “outliers” and were replaced by the mean of the condition (average % outliers: HV = 4.7, range: 4–5.1; PT = 4.2, range: 3.2–5.3). RTs ≤ 100 msec were considered as an error response.

### fMRI data acquisition and analysis

Siemens 3 T Verio with a 12 channel head coil. fMRI scans were carried out with gradient echo-planar-Imaging (EPI) sequence: TR = 2000 msec, TE = 25 msec, slice thickness = 4 mm, FOV = 240 mm, design matrix = 64 × 64, flip = 90^0^, and slices = 34. A rear-viewing reflecting mirror was mounted on the MR head coil facing a rear-projection screen placed at the back end of the scanner. A gradient echo EPI fieldmap was acquired for post-scan EPI distortion correction (TR = 1000 msec, TE1 = 3.97 msec, TE2 = 6.43 msec, FOV = 240 mm, slice thickness = 4 mm, design matrix = 64 × 64, flip = 55). T1-weighted anatomical image was acquired using the magnetization prepared rapid gradient echo (MPRAGE) sequence (TR = 3260 msec, TE = 2.26 msec, FOV = 256 mm, design matrix = 256 × 256, slice thickness = 1 mm, slices = 176).

fMRI data were processed and analyzed using the SPM12 software (the Wellcome Department of Imaging Neuroscience, University College London, London, UK). All EPI images were distortion corrected with a gradient echo EPI field-map collected during the fMRI session, and slice-timing corrected, realigned, and coregistered with the subject’s own high resolution T1 anatomical image. The DARTEL software and procedures were used to normalize the T1 and the EPI images to the MNI (Montreal Neurological Institute, Canada) template. At the first level analysis, the design matrix included seven response types (All-Go, SwGo, Switch, SSTGo, Stop-inhibit, Stop-respond, and the error response as a nuisance variable), Rest period, and 6 motion parameters as separate regressors. The efMRI activation was modeled using the canonical hemodynamic response function (HRF) with temporal and dispersion derivatives. The data were high-pass filtered at 128 sec and the epoch/event duration was set at 1 sec for the response types and 9 sec for the Rest period. Contrasts from the first level individual analysis were fed into the second (group) level analysis using the Flexible factorial design and t-tests. Sphericity for Subjects was set to be independent and for Response Type dependent. All statistical contrasts were corrected for multiple comparisons and all reported significant voxels survived a corrected multiple-comparison threshold of p < 0.05. For analysis showing activation clusters, a voxel level threshold of uncorrected p < 0.001 and a cluster threshold of FWE < 0.05 were applied^50^. For contrasts investigating the fronto-basal-ganglia response inhibition network, a single binary ROI mask was created with the WFU PickAtlas software (by the Functional MRI Laboratory at the Wake Forest University School of Medicine, CA). The ROI mask included the left M1 (LM1), right inferior-frontal cortex (rIFC), supplementary motor area (SMA), preSMA, and the basal ganglia. All activation loci reported in the study were verified using the Anatomy software^51,52^ and the WFU PickAtlas software with the Automated Anatomical Labeling^53,54^.

### Data Availability

All data generated or analyzed during this study are included in this published article.

## Electronic supplementary material

Supplementary Material
