# Supplementary material for: Lasting deficit in inhibitory control with mild traumatic brain injury
Source: Sci Rep. 2017 Nov 2;7:14902. doi: 10.1038/s41598-017-14867-y (PMC5668274; doi:10.1038/s41598-017-14867-y)
Supplement: Supplementary file 1 — Supplementary Material [file 41598_2017_14867_MOESM1_ESM.pdf]

Supplementary Material

Lasting deficit in inhibitory control with mild traumatic brain injury

Benjamin Xu[1,2], Marco Sandrini[1,2], Sarah Levy[2], Rita Volochayev[1], Oluwole Awosika[1], John A. Butman[2,3], Dzung L. Pham[2], Leonardo G. Cohen[1]

Affiliations:

1. Human Cortical Physiology and Neurorehabilitation Section, National Institute of Neurological Disorders and Stroke, National Institutes of Health, Bethesda, MD 20892, U.S.A.

2. Center for Neuroscience and Regenerative Medicine, Uniformed Services University of Health Sciences, Bethesda, MD 20814, U.S.A.

3. Radiology and Imaging Sciences, Clinical Center, National Institutes of Health, Bethesda, MD 20892, U.S.A.

***Corresponding author**:

Benjamin Xu, Ph.D.
The National Institutes of Health
5635 Fishers Ln
Rockville, MD 20852
Tel: 301-443-6545
Email: benxu1@mail.nih.gov

Table S1. TBI patients

Patient	Age	Sex	Education	Concussions	LOC	TTS (mon)	GCS	Lesion	MMSE	BDI-II
**PT1**	33	M	College	1	Maybe	34	14	L IT	29	24
**PT2**	24	F	College	3	<1 min	7	15	none	30	7
**PT3**	29	M	College	< 8	<10 min	41	15	none	29	7
**PT4**	21	M	High School	5	<1 min	97	15	L IT	29	11
**PT5**	32	F	College	1	<1 min	24	15	OFG	29	0
**PT6**	35	M	College	1	<1 min	12	15	none	29	9
**PT7**	31	F	College	1	<5 min	5	14	none	30	2
**PT8**	31	M	College	3	<1 min	100	15	none	25	16
**PT9**	27	M	College	<7	<3 min	9	none	none	29	16
**PT10***	23	F	College	1	<1 min	5	none	R IT	29	5
**PT11**	23	F	College	1	<1 min	24	none	none	29	5
**PT12**	28	M	College	1	<1 hr	16	none	none	30	0
**PT13***	36	M	College	1	<5 hrs	28	13	none	29	0
**PT14**	34	M	College	1	<20 min	8	none	R IT	30	1
**PT15**	23	M	College	1	<7 hrs	21	none	none	30	0
**PT16***	37	M	College	1	<1 hr	7	none	none	29	6
**PT17**	33	M	College	1	<5 min	38	none	R Precuneus	30	0

Note: LOC = lost of consciousness; TTS (mon) = time to study (in month) since last concussion; GCS = Glasgow Coma Scale; MMSE = Mini-mental State Examination; BDI-II = Beck Depression Inventory II. L = left; R = right; IT = inferior temporal gyrus; OFG = orbitofrontal gyrus. * = clinically diagnosed as moderate TBI. The behavioral results did not differ substantially when patients with moderate TBI, or with multiple concussions, or with brain lesions were excluded in the analysis (see Table 2 - 3 and Figure 1 below).

Table S2. Baseline memory and attention tests

a. All patients

Participants	Digit Span	CSM ACC %	M2S ACC %	CP RT/ACC %	PurT
**HC**	7.1 [±1]	91.3 [±8.2]	93.2 [±7.5]	522 [±102] / 85.8 [±15.8]	10.5 [±4.2]
**mTBI-all**	7.1 [±1]	88.6 [±12.5]	91.2 [±11.4]	554 [±106[/ 79.8 [±14.9]	10.4 [±4.8]
**mTBI-sc**	7.3 [±1]	86.3 [±13.8]	91 [±13]	576 [±99] / 82.1 [±14.2]	10.6 [±5.7]
**mTBI-nl**	7.1 [±1]	94.2 [±7.2]	89.5 [±12.3]	556 [±113] / 81.9 [±15.7]	11.1 [±5.8]
**mTBI-nm**	7 [±1]	88.3 [±13.8]	91.8 [±9.7]	551 [±116] / 81.5 [±13.4]	9.5 [±1.5]
	n/s	n/s	n/s	n/s	n/s

Notes: Results of two-sample t tests (healthy control [HC] vs mTBI patients [PT]). n/s = not significant; ANAM tests: CSM = Code Substitution (memory); M2S = Matching to Sample

(memory); CP = 1-back Continuing Performance (memory and attention); PurT = Pursuit Tracking (distance to target) (motor and attention); [ ] = standard deviation; mTBI-all = all patients; mTBI-sc = only patients with a single-reported concussion; mTBI-nl = excluded patients with lesions; mTBI-nm = excluded patients with moderate TBI.

Table S3. Stroop results

**Participants**	**Congruent** RT/Error(%)	**Incongruent** RT/Error(%)	**Control** RT/Error(%)	**Neutral** RT/Error(%)
**HC**	595[±81] / 1.2[±2.8]	737[±113] / 7.1[±7.1]	588[±71] / 1.2[±2.2]	642[±84] / 1.8[±3]
**mTBI-all**	637[±93] / 2.6[±3.6]	789[±80] / 20.3[±15.1]	647[±88] / 4.7[±6.5]	690[±86] /4.4[±5.6]
**mTBI-sc**	640[±92] / 2.5[±3.9]	799[±72] / 18.8[±15.7]	644[±66] / 3.3[±3.9]	692[±78] /3.8[±5.3]
**mTBI-nl**	619[±100] / 2.7[±3.4]	770[±90] / 17.3[±14.2]	623[±97] / 5[±7.4]	664[±90] /2.7[±4.1]
**mTBI-nm**	629[±93] / 2.5[±3.8]	781[±84] / 21.4[±13.5]	646[±95] / 5.4[±7]	683[±89] / 5.4[±5.7]

Note: Table 3 shows the results of the Stroop task with all HC and patients, and separate results from three sub-types of the TBI patients. Mix Repeated-measures ANOVAs (mRMANOVA) were performed for all patients combined (mTBI-all) and for the sub-types of patients (mTBI-sc, mTBI-nl, and mTBI-nm) separately. There were no significant main effect of Group (i.e., HC and PT) and interaction between Group and Stimulus Type (i.e., Congruent, Incongruent, Control, and Neutral) in the response time (RT). This is true when HC was compared separately with all three sub-types of patients. However, there were a significant main effect of Group and an interaction (Group x Stimulus Type) in response accuracy (ACC) regardless of whether or not patients were analyzed together or in separate sub-types (main effect and interaction: 1) HC vs mTBI-all: $F_{(1,32)} = 11.58$, $p < .002$; $F_{(3,96)} = 7.12$, $p < .001$; 2) HC vs mTBI-sc: $F_{(1,27)} = 8.29$, $p < .01$; $F_{(3,81)} = 5.03$, $p < .003$; 3) HC vs mTBI-nl: $F_{(1,26)} = 6.57$, $p < .05$; $F_{(3,78)} = 4.25$, $p < .01$; 4) HC vs mTBI-nm: $F_{(1,29)} = 14.6$, $p < .001$; $F_{(3,87)} = 8.46$, $p < .0001$). Post hoc Tukey comparisons ($p < .05$) showed that the interaction was mainly due to the significantly higher error rate of the patients relative to that of the HC. mTBI-all = all patients; mTBI-sc = only patients with a single-reported concussion; mTBI-nl = excluded patients with lesions; mTBI-nm = excluded patients with moderate TBI.

Figure S1. Results (All-Go and Switch conditions) of the 4CRT with sub-types of the TBI patients

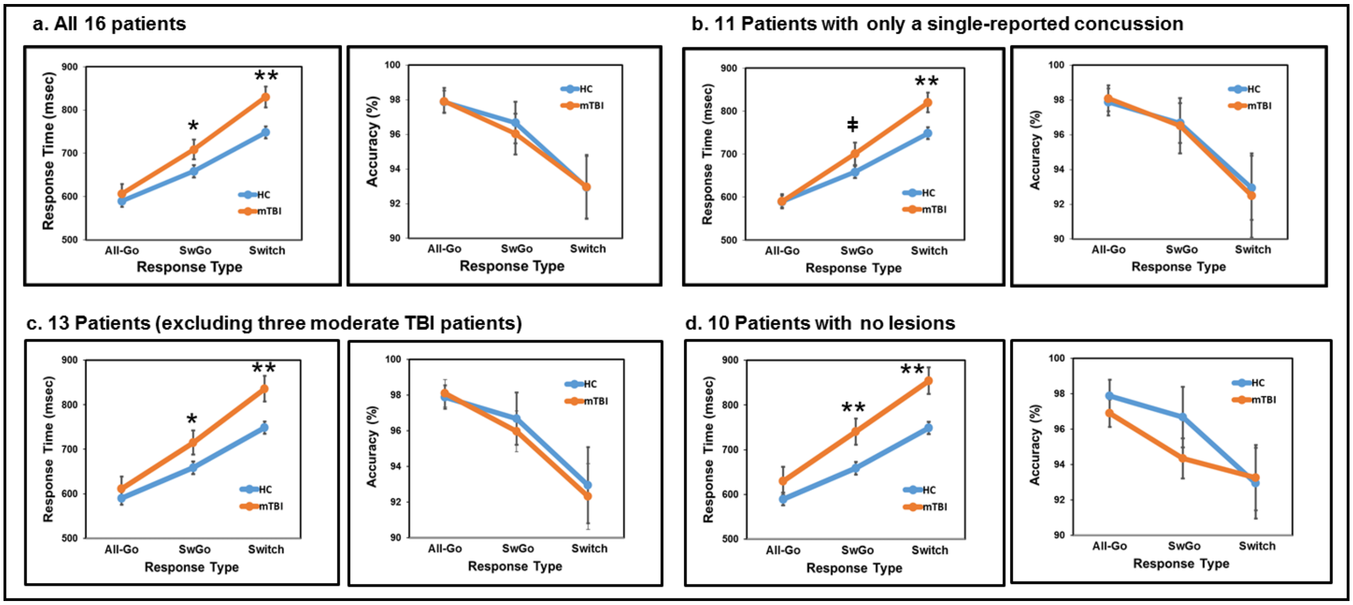

Figure 1 shows the results of the performance of the patients (PT) relative to the healthy controls (HC). Separate Mixed Repeated Measures ANOVA and planned one-sample t tests (one-tailed) were carried out for response time and accuracy of each of the sub-types of the TBI patients. All interactions between Response Time and Response Type, and between Accuracy and Response Type are statistically significant at $p < .01$. More detailed statistics for Figure 1a is reported in the main text of the manuscript. One patient's data were excluded in the analysis because the patient did not follow the instruction for performing the Switch task condition and the data were not usable. Note: All-Go = "go" response in the All-Go task condition; SwGo = "go" response in the Switch condition; Switch = the "Switch" response; * = $p < .05$; ** = $p < .001$; ǂ = $p < .07$.

Figure S2. fMRI activation during the 4CRT task

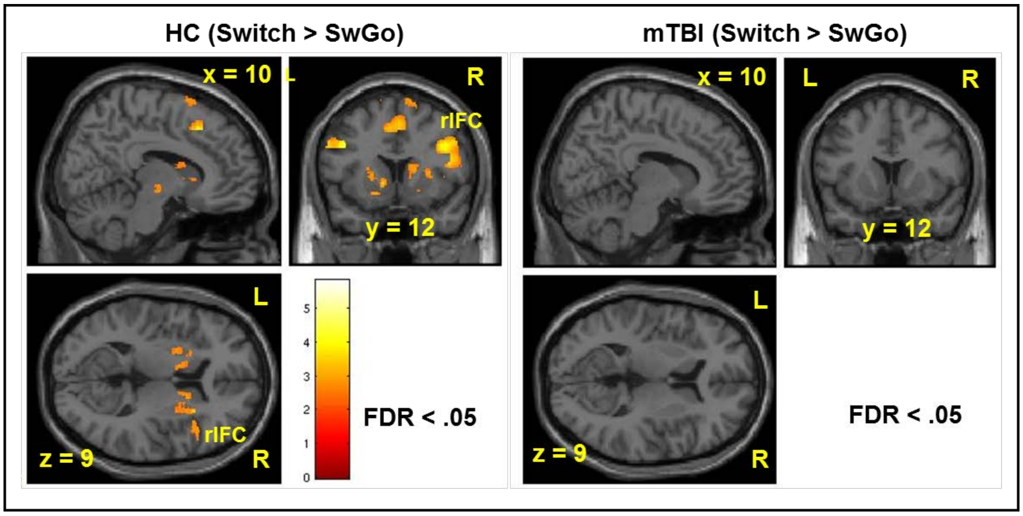

Figure 2. Brain regions showed significantly more activation in the fronto-basal-ganglia inhibitory network during the “Switch” response relative to the “go” response (SwGo) in the HC group. There was no significant difference between the “Switch” and SwGo activation in the mTBI group. The data were acquired using a binary regions-of-interest (ROI) mask that included LM1, rIFC, SMA, preSMA, and the basal ganglia that were part of the fronto-basal-ganglia response inhibition network. All significant voxels survived corrections for multiple comparisons using FDR < .05; Ext = 20.

Figure S3. fMRI whole-brain analysis

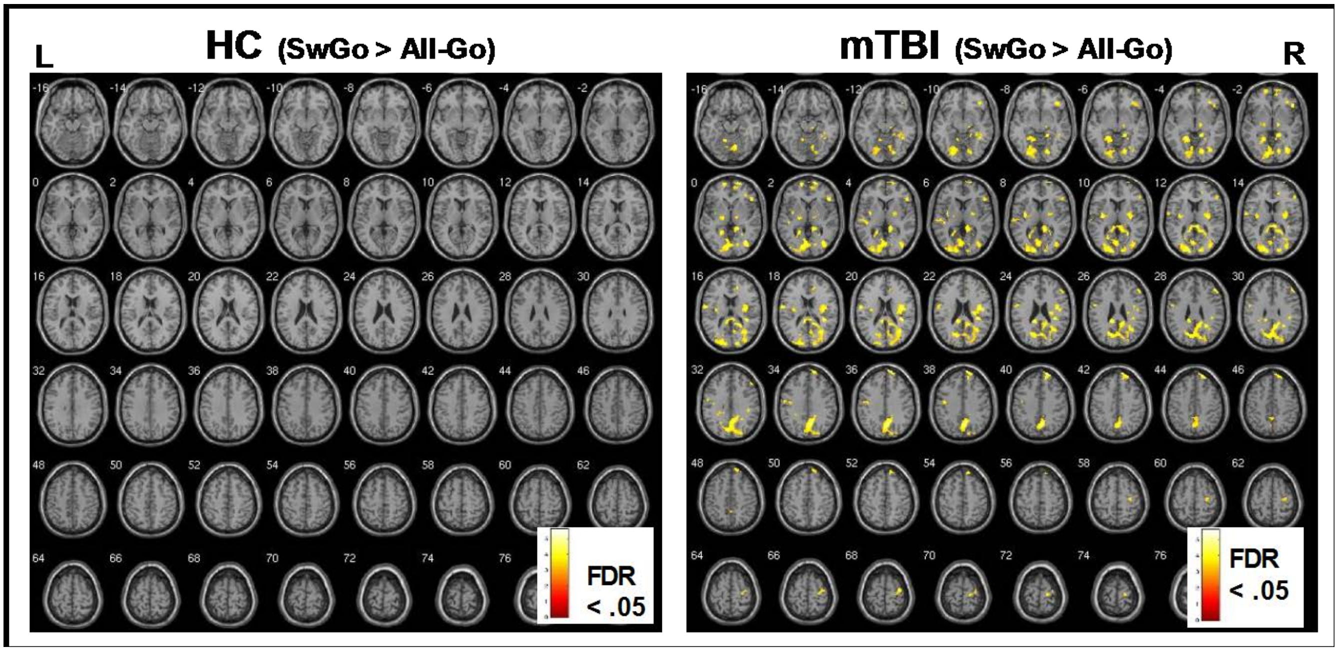

Figure 3. Activation during the “go” responses (SwGo) in the Switch condition when 25% of the “go” response must be inhibited for a correct Switch response. Relative to the All-Go (i.e., the

"go" responses in the All-Go condition), the mTBI group recruited more extensive brain regions than the HC group. Threshold (SwGo-All-Go) FDR < .05, Ext = 100.

Figure S4.

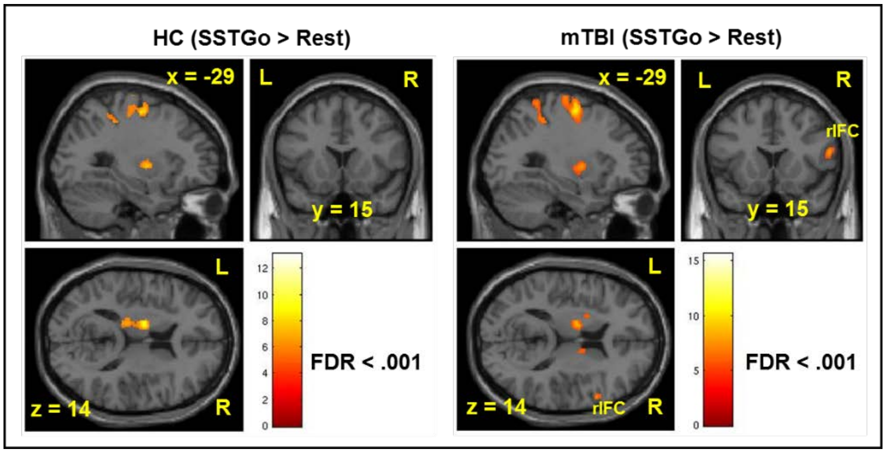

Figure 4. Whole brain analysis of fMRI activation during the "go" responses in the Stop-signal condition when 25% of the primary "go" responses must be stopped/inhibited. Eleven HC and 11 mTBI patients who successfully stopped (i.e., stop-inhibit response) about 50% of the time when a stop-signal appeared were included in the analysis, a standard requirement for estimating the stop-signal response time (SSRT) [1]. Relative to Rest, the mTBI group recruited more extensive brain regions than the HC group in the right inferior-frontal cortex (rIFG) and the striatum brain regions that have been shown to be critical for inhibitory control including response switching [2,3]. Threshold FDR < .001, Ext = 100. SSTGo = the "go" response in the Stop-signal task condition.

References

1 Logan, G. D., Cowan, W. B. & Davis, K. A. On the ability to inhibit simple and choice reaction time responses: a model and a method. J Exp Psychol Hum Percept Perform 10, 276-291 (1984).

2 Xu, B. et al. Effect of foreknowledge on neural activity of primary "go" responses relates to response stopping and switching. Frontiers in human neuroscience 9, 34, doi:10.3389/fnhum.2015.00034 (2015).

3 Kenner, N. M. et al. Inhibitory motor control in response stopping and response switching. J Neurosci 30, 8512-8518, doi:10.1523/JNEUROSCI.1096-10.2010 (2010).
